# Neutralizing Activity of Anti-interferon-γ Autoantibodies in Adult-Onset Immunodeficiency Is Associated With Their Binding Domains

**DOI:** 10.3389/fimmu.2019.01905

**Published:** 2019-08-14

**Authors:** Umpa Yasamut, Weeraya Thongkum, Sutpirat Moonmuang, Supachai Sakkhachornphop, Romanee Chaiwarith, Jutarat Praparattanapan, Jiraprapa Wipasa, Kriangkrai Chawansuntati, Khuanchai Supparatpinyo, Ethan Lai, Chatchai Tayapiwatana

**Affiliations:** ^1^Division of Clinical Immunology, Department of Medical Technology, Faculty of Associated Medical Sciences, Chiang Mai University, Chiang Mai, Thailand; ^2^Faculty of Associated Medical Sciences, Center of Biomolecular Therapy and Diagnostic, Chiang Mai University, Chiang Mai, Thailand; ^3^Ph.D. Program in Biomedical Science, Faculty of Associated Medical Sciences, Chiang Mai University, Chiang Mai, Thailand; ^4^Faculty of Medicine, Research Institute for Health Sciences, Chiang Mai University, Chiang Mai, Thailand; ^5^Department of Internal Medicine, Faculty of Medicine, Chiang Mai University, Chiang Mai, Thailand; ^6^Pall Filtration, Singapore, Singapore

**Keywords:** interferon-γ, anti-interferon-γ autoantibody, neutralizing antibody, competitive-binding ELISA, adult-onset immunodeficiency

## Abstract

Adult-onset immunodeficiency (AOID) with anti-interferon-γ (IFN-γ) autoantibodies (autoAbs) is an emerging immunodeficiency syndrome in Asian countries. The presence of neutralizing anti-IFN-γ autoAbs are significantly associated with severe disseminated opportunistic infections. However, the characteristics of the neutralizing antibodies in patients are poorly defined. To better understand the properties of the anti-IFN-γ autoAbs in patients with opportunistic infections, a simplified competitive-binding ELISA was developed. The domains recognized by anti-IFN-γ autoAbs were assessed based on their competition with commercial neutralizing mouse anti-IFN-γ monoclonal antibodies (mAbs). First, the binding affinity and neutralizing capacity of these mAbs (clones B27, B133.5, and MD-1) were characterized. Kinetic analysis and epitope binning using bio-layer interferometry showed the comparable binding affinity of these mAbs to full-length IFN-γ and to the adjacent binding region. These mAbs did not recognize the synthetic 20-mer peptides and inhibited IFN-γ-mediated functions differently. In a competitive-binding ELISA, the anti-IFN-γ autoAbs in AOID serum blocked B27, B133.5, and MD-1 mAb binding. This evidence suggested that the autoAbs that competed with neutralizing mouse anti-IFN-γ mAbs recognized a discontinuous epitope of homodimeric IFN-γ as these mAbs. The patient autoAbs that recognized the B27 epitope exhibited strong neutralizing activity that was determined by the functional analysis. Our results demonstrated the heterogeneity of the autoAbs against IFN-γ in AOID patients and the different patterns among individuals. These data expand upon the fundamental knowledge of neutralizing anti-IFN-γ autoAbs in AOID patients.

## Introduction

Interferon-γ (IFN-γ) is a pleiotropic cytokine that is mainly produced by T lymphocytes and natural killer cells. IFN-γ potently promotes antimicrobial responses, antigen processing, inflammation, macrophage (M1 subtype) differentiation, growth suppression, cell death, tumor immunity, and autoimmunity (1). The IFN-γ receptor consists of two subunits, interferon-γ receptor 1 (IFNGR1) and interferon-γ receptor 2 (IFNGR2), which interact with a family of non-receptor protein tyrosine kinases, the Janus activated kinase (JAK) family. The binding of an IFN-γ homodimer to its receptor activates JAK-STAT1 signaling. Phosphorylated STAT1 homodimers translocate to the nucleus and bind to the gamma activating sequence (GAS) to regulate the gene expression of the IFN-γ-stimulated genes. In the context of immunity to intracellular pathogens, IFN-γ is involved in regulating the Toll-like receptor (TLR) pathway, activating a GTPase family member that mediates host resistance to intracellular microbes, inducing ROS and RNI production, increasing antigen presentation, upregulating MHC class I and MHC class II expression, and modulating interleukin 12 (IL-12) production to amplify IFN-γ signaling (2). Therefore, the IL-12-dependent IFN-γ axis has an important role in controlling infection with intracellular microbes, especially mycobacteria, and Salmonella.

Genetic defects in the IL-12-dependent IFN-γ pathway cause both early-onset and late-onset immunodeficiency. Mendelian susceptibility to mycobacterial diseases (MSMD) is considered a genetic defect that can result in severe, persistent, or recurrent non-tuberculous mycobacterial (NTM) or Salmonella infections in children (3). Inherited defects, including IFN-γ receptor deficiency, IL-12 cytokine/receptor deficiency, and downstream signaling (STAT1 and IRF8) defects, have been reported. In late childhood or adulthood, GATA2 deficiency gives rise to disseminated infection. In addition to genetic defects, the association of anti-IFN-γ autoAbs with disseminated opportunistic infections has been reported (4–6).

Anticytokine autoantibodies have been identified as a cause of immunodeficiency in previously healthy adults. Currently, the presence of neutralizing anti-IFN-γ autoAbs is associated with disseminated NTM infections or infections with other intracellular pathogens in patients who have no HIV infection or other types of immunosuppression. This syndrome is classified as adult-onset immunodeficiency (AOID). The number of such cases in Asian countries has increased over time (4–6). Several studies have identified the presence of anti-IFN-γ autoAbs in these patients and have demonstrated that these antibodies inhibit the downstream signaling of IFN-γ (4, 7, 8). The properties of these autoAbs were investigated to understand the characteristics of the pathogenic antibodies. It has been reported that the anti-IFN-γ autoAbs in these patients were of the IgG1 and IgG4 subclasses. In ~40% of patients, these autoAbs recognize the linear epitope containing the KRKR motif, which is located at the C-terminus of IFN-γ (9). In some patients, neutralizing anti-IFN-γ autoAbs did not recognize the C-terminal epitope, suggesting that those autoAbs recognize other epitopes that are essential for IFN-γ-mediated signaling (8, 9).

In 1989, mouse mAbs against recombinant human IFN-γ were generated and characterized. Five of these antibodies (B22, B27, 35, 32, and 3-6) were selected for structure-based functional analysis (10). The data demonstrated that the 3–6 mAb, which binds an epitope located on the 15 C-terminal amino acids, does not block IFN-γ bioactivity. In addition, the B22, and B27 mAbs, which recognize a nearby epitope constructed by dimeric IFN-γ, inhibit the functional activity of IFN-γ, and the B27 mAb has a strong anti-viral activity compared with that of other mAbs (10). The results suggested that the B22/B27 epitope is associated with IFN-γ-mediated functions; thus, mAbs that recognize this epitope have neutralizing ability. Previously, autoAb clones that recognized the KRKR motif at the C terminus of IFN-γ have been reported in AOID patients with mycobacterial disease (7). Nonetheless, no evidence has demonstrated that there are autoAb clones that recognize the conformational epitope that is crucial for IFN-γ-mediated functions. In this study, a neutralizing anti-IFN-γ autoAb that recognized the discontinuous epitope was identified in AOID patients with a competitive-binding ELISA using B27, B133.5, and MD-1 mAbs. This finding indicated that the neutralizing anti-IFN-γ autoAbs in AOID patients were heterogeneous and that the level of each autoAb clone differed among individuals.

## Materials and Methods

### Serum Samples

In this pilot study, nine non-HIV adult patients with opportunistic infections were enrolled. These patients had no history of immunosuppression and were classified as having (AOID). Patient sera were randomly selected based on the presence of anti-IFN-γ Abs. Four male and 5 female patients aged 43–59 years with experienced with at least one episode of NTM or related opportunistic infections. Normal sera (*n* = 3) from healthy individuals (aged 47–54 years) who had no infections or immunosuppressive status were included for comparison. The sample collection was approved by the ethics committees of the Faculty of Medicine (Project No. 105/2557) and the Research Institute for Health Sciences of Chiang Mai University (Project No. 13/56). Ethics approval and informed consent from participants were obtained in accordance with the guidelines of the Helsinki declaration.

### Antibodies and Recombinant IFN-γ

For ELISA and functional assays, monoclonal antibodies against the IFN-γ clones B27 and B133.5 were obtained from ImmunoTools in Germany, and clone MD-1 was purchased from BioLegend Inc. in CA. These antibodies were IgG1-specific to IFN-γ and exhibited neutralizing capacity against IFN-γ (10–12). For the neutralization assay, mouse IgG1 (clone MOPC-21) (BioLegend Inc.) was used as the isotype-matched antibody control. For flow cytometry, FITC-conjugated anti-human HLA-DR and DP (clone HL-38) and isotype-matched control FITC-conjugated mouse IgG2a, were purchased from ImmunoTools in Germany. PE-conjugated anti-human phospho-STAT1 (pY701) antibody (BD Pharmingen) was purchased from BD Biosciences. Recombinant human IFN-γ (rIFN-γ) was purchased from R&D Systems in MN.

### Generation of H6-Tagged Recombinant Human IFN-γ

The DNA fragment encoding human IFN-γ (hIFN-γ) was amplified from pGEM-IFNG (Sino Biological Inc., Beijing, China) by polymerase chain reaction (PCR) using primers with a 6xHis-tag. The PCR product was further purified using a GeneJET PCR purification kit (Thermo Fisher Scientific). The hIFNG-H6 gene was subsequently cloned into the pPET21a expression plasmid using the flanking NheI and HindII sites. The ligation product was transformed into competent *Escherichia coli* BL21 (DE3) cells to produce the recombinant hIFN-γ-H6 protein. The recombinant proteins were purified by affinity chromatography on a HisTrap column using ÄKTA Prime plus (GE Healthcare, Piscataway, NJ). SDS-PAGE and Pageblue staining were used to determine the purity of the rIFN-γ-H6 protein. This rIFN-γ-H6 was used to determine the binding affinity of mouse anti-IFN-γ mAbs by bio-layer interferometry (BLI).

### Detection of Human Anti-IFN-γ autoAbs by Indirect ELISA

Microtiter plates were coated with 50 μl of rIFN-γ in bicarbonate buffer (pH 9.6) per well and incubated overnight at 4°C in a humidified chamber. The following steps were performed at room temperature. The coated wells were washed four times with 0.05% Tween 20 in phosphate-buffered saline (PBS) and blocked with 200 μl of a blocking solution (PBS with 2% skim milk) for 1 h. After washing twice, 50 μl of patient serum (diluted 1:2,500 in blocking solution) was added and incubated for 1 h. After washing four times, 50 μl of horseradish peroxidase (HRP)-conjugated rabbit anti-human immunoglobulin G (IgG) (SeraCare, Milford, MA) that was diluted 1:5,000 was added and incubated for 1 h. The reactions were developed with a chromogenic substrate, 3,3′,5,5′-tetramethylbenzidine (TMB) substrate and stopped with 1 N hydrochloric acid (HCl). The absorbance was measured at 450 nm with an ELISA reader.

### Determination of the Binding Activity of Mouse Anti-IFN-γ mAbs by Indirect ELISA

To confirm the binding of the three commercial monoclonal antibodies including B27, B133.5, and MD-1 to IFN-γ, an indirect ELISA was performed. Microtiter plates were coated with 50 μl of rIFN-γ in bicarbonate buffer (pH 9.6) per well and incubated overnight at 4°C in a humidified chamber. The following steps were performed at room temperature. The coated wells were washed four times with 0.05% Tween 20 in PBS and blocked with 200 μl of a blocking solution (PBS with 2% skim milk) for 1 h. After washing twice, 50 μl of 0.3 μg/ml of each mAb was added and incubated for 1 h. After washing four times, 50 μl of HRP-conjugated goat anti-mouse immunoglobulin (dilution 1:3,000) was added and incubated for 1 h. The reactions were developed with TMB substrate and stopped with 1 N HCl. The absorbance was measured at 450 nm with an ELISA reader.

### Binding Affinity and Epitope Binning of Mouse Anti-IFN-γ mAbs Using Bio-Layer Interferometry (BLI)

Kinetics of mouse anti-IFN-γ mAbs (B27, B133.5, and MD-1) were analyzed using a ForteBio Octet RED96e instrument. All the assays were performed at 200 μl/well in 0.05% Tween 20-PBS at 30°C. Ten micrograms per milliliter of His-tagged IFN-γ was loaded onto the surface of anti-penta His biosensors (HIS1K), followed by a 120 s biosensor washing step. The association of IFN-γ on the biosensor to the individual mAbs in solution was analyzed for 120 s. The dissociation of the interaction was probed for 600 s. The K_D_ was calculated using the ratio k_d_/k_a_.

For epitope binning, His-tagged IFN-γ was captured onto anti-penta His biosensors and was coated with testing mAbs (antibody bin) at a saturating concentration. The epitopes of the other mAbs were probed in relation to the testing mAbs. Raw data were processed using the ForteBio Data Analysis Software, and the antibody pairs were assessed for competitive binding. Additional binding by the second antibody indicated an unoccupied epitope (non-competitor), while no binding indicated epitope blocking (competitor).

### Detection of Neutralizing Human Anti-IFN-γ autoAbs by Flow Cytometry

To determine the neutralizing activity of anti-IFN-γ autoAbs in patient sera on IFN-γ signaling, the inhibition of IFN-γ-induced STAT1 phosphorylation and IFN-γ-induced MHC-II expression were observed. For intracellular staining of phosphorylated STAT1 (pSTAT1), a monocytic cell line, THP-1 cells were stimulated for 15 min at 37°C with rIFN-γ pre-incubated with patient serum or media alone as baseline. After stimulation, the cells were fixed by adding formaldehyde to obtain a final concentration of 1.5%. Then, the cells were incubated in fixative for 10 min at room temperature. After pellet the cells by centrifugation, the cells were permeabilized with 500 μl of ice-cold absolute methanol and incubated at 4°C for 10 min. After washing and blocking, the cells were then stained with PE-conjugated anti-human phospho-STAT1 (pY701) antibody for a further 30 min on ice in the dark. After washing, the cells were resuspended in PBS. Data were collected with a CyAn ADP flow cytometer (Beckman Coulter) and analyzed with Kaluza software (Beckman Coulter).

For the IFN-γ-induced MHC-II expression experiment, rIFN-γ was incubated with patient serum in 10% FBS-RPMI supplemented with penicillin and streptomycin for 1 h at 37°C before stimulation. THP-1 cells (4 ×10^5^ cells) were stimulated with 400 μl of 50 ng/ml of rIFN-γ in the absence or presence of patient serum (final dilution 1:200) for 24 h at 37°C in a 5% CO_2_ incubator. At 24 h post-treatment, the cells were harvested to detect the surface expression of MHC-II by flow cytometry. The cells were washed with PBS three times and blocked with 50 μl of 10% heat-inactivated AB serum in PBS for 30 min. For MHC-II staining, 2.5 μl of FITC-conjugated anti-human HLA-DR and DP or the isotype-matched antibody control were added and incubated for 30 min on ice. After washing, the cells were resuspended in 1% paraformaldehyde-PBS. The data were collected with a FACSort flow cytometer (BD Bioscience).

### Detection the Neutralizing Activity of Mouse Anti-IFN-γ mAbs by Flow Cytometry

To determine the neutralizing activity of mouse anti-IFN-γ mAbs, rIFN-γ was incubated with each mAb for 1 h at 37°C. THP-1 cells were stimulated with 400 μl of 50 ng/ml of rIFN-γ in the absence or presence of a mAb (final concentration 1 μg/ml). After 24 h, cells were harvested for MHC-II staining as described above.

### Competitive-Binding ELISA for the Detection of Anti-IFN-γ autoAbs in AOID Patients

To determine the anti-IFN-γ autoAbs recognizing the corresponding domain of neutralizing mAbs, a competitive-binding ELISA was developed. Microtiter plates were coated with 50 μl of rIFN-γ in bicarbonate buffer (pH 9.6) per well and incubated overnight at 4°C in a humidified chamber. The following steps were performed at room temperature. The coated wells were washed four times with 0.05% Tween 20 in PBS and blocked with 200 μl of a blocking solution (PBS with 2% skim milk) for 1 h. The mAbs (B27, B133.5, or MD-1) (final concentration 0.3 μg/ml) were mixed individually with patient sera (final dilution 1:100) before being added to the IFN-γ-coated wells. After removing the blocking solution and washing, the mAb-IFN-γ mixture was added and incubated for 1 h. After washing four times, 50 μl of HRP-conjugated goat anti-mouse immunoglobulin (diluted 1:3,000 in PBS with 2% skim milk and 1% human serum) was added and incubated for 1 h. The reactions were developed with TMB substrate and stopped with 1 N HCl. The absorbance was measured at 450 nm with an ELISA reader.

### Epitope Mapping With IFN-γ Peptides by Indirect ELISA

To identify an epitope that was recognized by mouse anti-IFN-γ mAbs, indirect ELISA was performed. Microtiter plates were coated with 50 μl of streptavidin (2 μg/ml) in bicarbonate buffer (pH 9.6) per well and incubated overnight at 4°C in a humidified chamber. The other steps were performed at 37°C in a humidified chamber. The coated wells were washed four times with 0.05% Tween 20 in PBS. Each peptide (2 μg/ml) was added and incubated for 1 h. After washing, non-specific binding was blocked with a blocking solution (PBS with 2% skim milk). The mAbs (B27, B133.5, or MD-1) (final concentration 0.3 μg/ml) were added and incubated for 1 h. After washing four times, 50 μl of HRP-conjugated goat anti-mouse immunoglobulin (dilution 1:3,000) was added and incubated for 1 h. The reactions were developed with TMB substrate and stopped with 1 N HCl. The absorbance was measured at 450 nm with an ELISA reader. To identify an epitope that was recognized by human anti-IFN-γ autoAbs, AOID serum (dilution 1:100) and HRP-conjugated rabbit anti-human IgG (dilution 1:5,000) were used.

## Results

### Measurement of Anti-IFN-γ autoAbs in AOID Patient Sera

**Figure 1:**
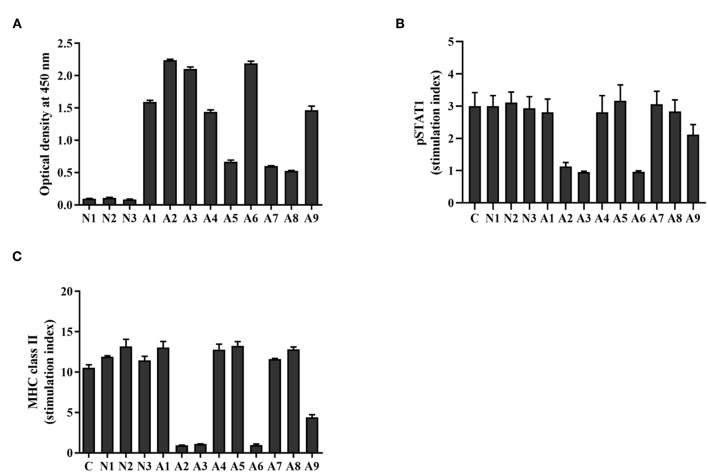
Level and neutralizing ability of anti-IFN-γ autoAbs in AOID sera. **(A)** Anti-IFN-γ autoAbs in patient sera [cohort; patients with opportunistic infections (*n* = 9) and healthy controls (*n* = 3)] were measured by indirect ELISA. The neutralizing capacity of anti-IFN-γ autoAbs was assessed with a cell-based assay. THP-1 cells were treated with IFN-γ in the presence or absence of patient serum. **(B)** The intracellular pSTAT1 and **(C)** the surface MHC-II expression was evaluated by flow cytometry. The stimulation index was the ratio of the fluorescence intensity of those proteins in stimulated cells divided by that in unstimulated cells. Experiments were performed in triplicate. Error bars represent the mean ± SEM.

The presence of anti-IFN-γ autoAbs from the sera of AOID patients (*n* = 9) and healthy controls (*n* = 3) were determined by indirect ELISA. Anti-IFN-γ autoAbs were detected in the AOID patients but not in the healthy controls, as shown in Figure 1A. From the nine AOID samples, the patients could be categorized into three groups based on the optical density at 450 nm: OD450 nm > 0.5 but <1.0 (*n* = 3), OD450 nm ≥1.0 but <2.0 (*n* = 3), and OD450 nm ≥2.0 (*n* = 3). These data suggest that the levels of anti-IFN-γ autoAbs among the patients were different. Apart from that, the diversified affinity of polyclonal antibodies from each serum sample influenced the absorbance level at OD450 nm.

### Investigation of Neutralizing Anti-IFN-γ autoAbs in AOID Patient Sera

To assess the neutralizing capacity of anti-IFN-γ autoAbs in AOID patients, functional analysis was performed by measuring the IFN-γ-induced STAT1 phosphorylation, and the IFN-γ-mediated MHC-II expression. After stimulation with rIFN-γ, the intracellular pSTAT1 level, and the surface expression of MHC-II in THP-1 cells was found to have increased in comparison with that in unstimulated cells, as demonstrated by the stimulation index. In the presence of neutralizing antibodies in the patient serum, the stimulation index decreased. The results show that the serum from four patients reduced the stimulation index of pSTAT1 and MHC-II at the measured dilution (dilution 1:200) (Figures 1B,C, Supplementary Figure S1). The pSTAT1 level is concordant with MHC class II expression level. Patient sera with OD450 nm ≥2.0 by indirect ELISA (A2, A3, and A6) inhibited the level of pSTAT1 and MHC-II ~65 and 90%, respectively. One of sample with OD450 nm ≥1.0 but <2.0 (A9) inhibited those markers ~30 and 58%, respectively. The serum from the other five samples did not exhibit neutralizing activity at the measured dilution. These data imply that the neutralizing activity of anti-IFN-γ autoAbs in AOID sera among the patients was distinct. The neutralizing activity was presumably relied on the Ab populations in serum samples which inherit the different degree of binding affinity against the neutralizing epitopes.

### Binding and Neutralizing Activity of Mouse Anti-IFN-γ mAbs

**Figure 2:**
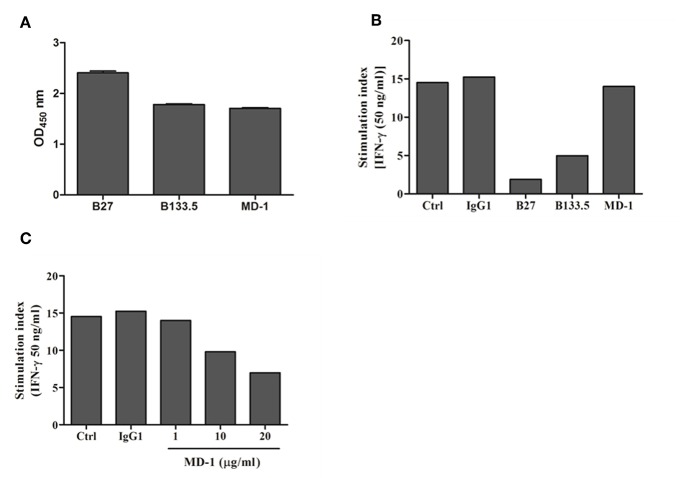
Binding and neutralizing activity of mouse anti-IFN-γ mAbs against IFN-γ. **(A)** The binding activity of the mouse mAbs was determined by indirect ELISA in triplicate. Error bars represent the mean ± SEM. **(B,C)** Neutralizing activity of the mouse anti-IFN-γ mAbs was assessed by a cell-based assay. THP-1 cells were treated with IFN-γ in the presence or absence of each mAb. The surface MHC-II expression was evaluated by flow cytometry, and the stimulation index was determined by calculating the stimulated/unstimulated ratio.

**Figure 3:**
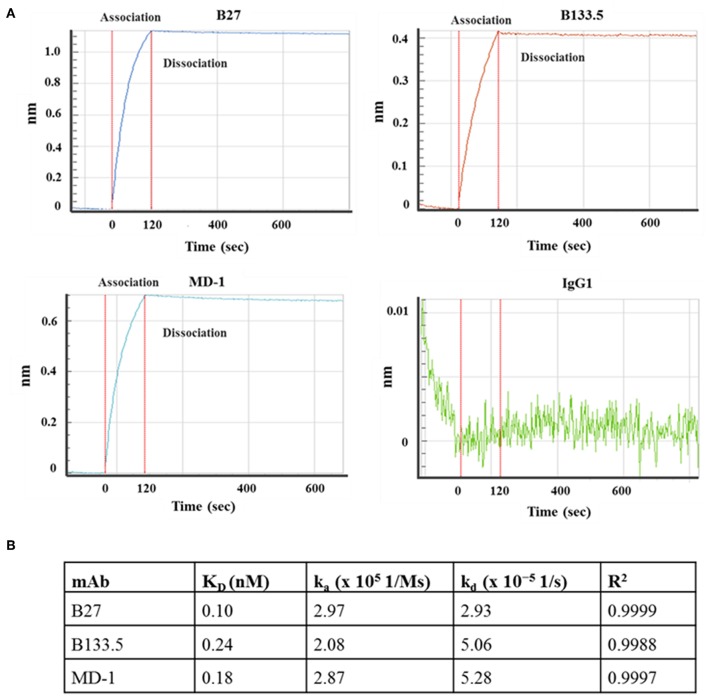
Kinetic analysis of the mouse anti-IFN-γ mAbs. **(A)** Sensograms show the association and dissociation curve of the mAb clones B27, B133.5, and MD-1. **(B)** Association rate constant (k_a_), dissociation rate constant (k_d_), and affinity (K_D_) of each mAb.

**Figure 4:**
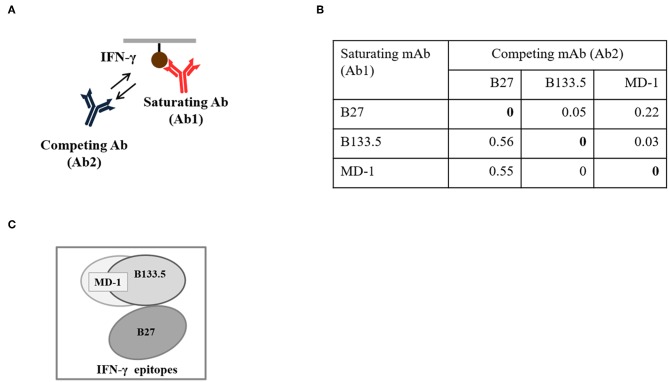
Epitope binning of the mouse anti-IFN-γ mAbs. **(A)** Diagram of the binning analysis. **(B)** Binning analysis of the mouse anti-IFN-γ mAbs clones B27, B133.5, and MD-1. **(C)** Clustered epitopes from epitope binning study.

In this study, three commercial neutralizing anti-IFN-γ mAbs were selected for determining the binding location of anti-IFN-γ autoAbs by a competitive-binding ELISA. As this purpose, the binding activity and the neutralizing capacity of the commercial mAbs were confirmed before use. The binding activity of the three mAbs (clone B27, B133.5, and MD-1) was confirmed by indirect ELISA. The optical density values obtained with B27, B133.5, and MD-1 were 2.41, 1.78, and 1.71, respectively (Figure 2A). To evaluate the neutralizing capacity of these mAbs, MHC-II expression mediated by IFN-γ was analyzed by flow cytometry. The percentages of inhibition in the presence of B27, B133.5, and MD-1 (1 μg/ml) were ~80, 60, and 5%, respectively (Figure 2B), and in the case of MD-1 (10 μg/ml), it was ~50% (Figure 2C). In addition, the binding affinity of each mAb was determined by monitoring the binding kinetics as shown in Figures 3A,B. The epitope recognized by each mAb was clustered and is demonstrated in Figures 4A–C. The results suggested that the B27 mAb recognizes a distinct epitope from those of the B133.5 and MD-1 mAbs, whereas B133.5 binds to a similar or overlapping epitope as that targeted by MD-1. Based on these results, it is evident that the different mAbs exhibited different neutralizing activities. Taken together, the results suggest that B27, B133.5, and MD-1 mAbs recognize different epitopes and exhibit different neutralizing capacities.

### Development of a Competitive-Binding ELISA to Detect the Interacting Domains of Anti-IFN-γ autoAbs in AOID Patients

**Figure 5:**
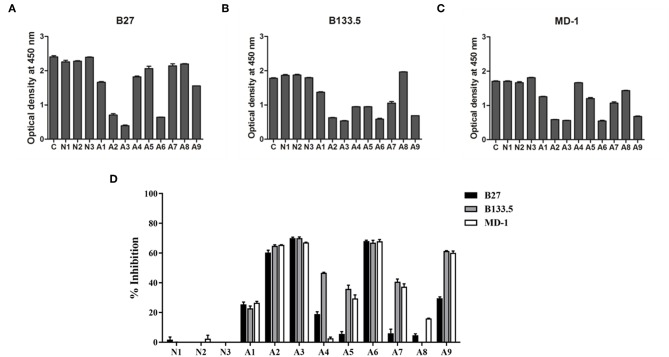
Characterization of anti-IFN-γ autoAbs in AOID patients by competitive-binding ELISA. The domains recognized by anti-IFN-γ autoAbs in patient sera were evaluated by competitive-binding ELISA. The binding of each mouse neutralizing anti-IFN-γ mAb (B27, B133.5, and MD-1) was competed with the anti-IFN-γ autoAbs in each patient serum. The reduction of the optical density demonstrates that there was competition. **(A)** The optical density of the B27 mAb. **(B)** The optical density of the B133.5 mAb. **(C)** The optical density of the MD-1 mAb. **(D)** Percentage of inhibition was calculated and showed the relative level of anti-IFN-γ autoAbs recognizing the mAb-binding epitope in patient sera. Experiments were performed in triplicate. Error bars represent the mean ± SEM. C, non-competitive condition; N, normal serum; A, AOID patient serum.

The domains for anti-IFN-γ autoAbs binding were assessed by competitive-binding ELISA. The sera were individually combined with each of the three mAbs before being added to IFN-γ-coated ELISA plates. The binding signal of the mAb in the competitive condition was compared with that in the non-competitive condition (Figures 5A–C) and calculated as the percentage of inhibition (Figure 5D). The results show that there were different patterns of inhibition among individuals. The percentages of inhibition in three of the samples (A2, A3, and A6) were ~60%, and A1 exhibited ~20% inhibition, which was found to be in competition with all the mAbs. For the other samples, the percentage of inhibition with the B27 mAb was low, but the percentage of inhibition was in the range of 40–60% for the B133.5 or MD-1 mAbs. These results suggest that anti-IFN-γ autoAbs in patients interact with the determinants recognized by neutralizing anti-IFN-γ mAbs and exhibit different patterns of autoAb population.

### Identification of the Epitope Recognized by the Neutralizing Anti-IFN-γ Antibodies

**Figure 6:**
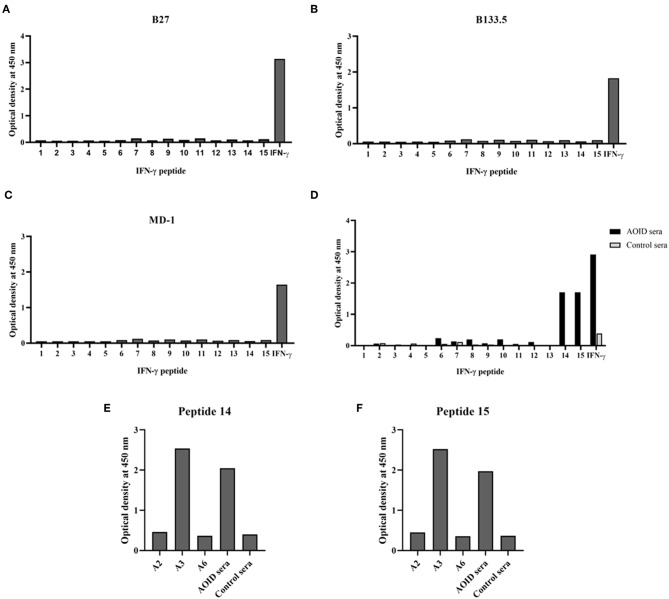
Identification of the epitope recognized by neutralizing anti-IFN-γ mAbs and autoAbs using biotinylated peptides and ELISA. **(A)** Epitope mapping of the mouse anti-IFN-γ mAb clone B27, **(B)** the mAb clone B133.5, and **(C)** the mAb clone MD-1. **(D)** Epitope mapping of the human anti-IFN-γ autoAbs in AOID patients, 10 serum samples from individuals with AOID, or 10 serum samples from healthy controls were pooled and tested. Epitope mapping of three of the serum samples from individuals with AOID that had strong neutralizing capacity using **(E)** peptide 14 and **(F)** peptide 15.

To identify the epitopes recognized by the mouse anti-IFN-γ mAbs, epitope mapping using peptides was performed by ELISA. The 15 biotinylated peptides from human IFN-γ that were generated as shown in Table 1. Three of the mouse anti-IFN-γ mAbs, including the clones B27, B133.5, and MD-1, were analyzed. The results showed that B27, B133.5, and MD-1 did not recognize these peptides (Figures 6A–C). Epitope mapping of human anti-IFN-γ autoAbs in the pooled AOID sera revealed that those antibodies bind to peptides 14 and 15 (Figure 6D). Individual sera with strong neutralizing activity, including sample A2, A3, and A6, were further examined. The anti-IFN-γ autoAbs in sample A3 recognized peptides 14 and 15, whereas those in samples A2 and A6 did not (Figures 6E,F). Taken together, the autoAbs in some AOID patient sera recognized non-linear epitopes.

**Table 1:** Amino acid sequence and position of peptides corresponding to human IFN-γ (9).

**Peptide**	**Position^a^**	**Amino acid sequence^b^**
1	−13 to 7	QLCIVLGSLGCYCQDPYVKE
2	−3 to 17	CYCQDPYVKEAENLKKYFNA
3	8–27	AENLKKYFNAGHSDVADNGT
4	18–37	GHSDVADNGTLFLGILKNWK
5	28–47	LFLGILKNWKEESDRKIMQS
6	38–57	EESDRKIMQSQIVSFYFKLF
7	48–67	QIVSFYFKLFKNFKDDQSIQ
8	58–77	KNFKDDQSIQKSVETIKEDM
9	68–87	KSVETIKEDMNVKFFNSNKK
10	78–97	NVKFFNSNKKKRDDFEKLTN
11	88–107	KRDDFEKLTNYSVTDLNVQR
12	98–117	YSVTDLNVQRKAIHELIQVM
13	108–127	KAIHELIQVM AELSPAAKTG
14	118–143	AELSPAAKTGKRKRSQMLFRGRRASQ
15	128–143	KRKRSQMLFRGRRASQ

*The formerly identified KRKR motif (7) is shared by peptide 14 and 15 (marked in red color)*.

a *Mature IFN-γ comprised 143 amino acids. Peptides 1 and 2 included some of the amino acids of the signal peptide in the IFN-γ precursor*.

b *The peptides were 20 amino acids in length with 10 amino acids overlapping except for peptide 14*.

## Discussion

Patients with anti-IFN-γ autoAbs have impaired IFN-γ signaling, which contributes to severe disseminated infection from intracellular pathogens. Importantly, the presence of neutralizing antibodies but not of non-neutralizing antibodies is associated with disseminated infection with NTM. It has been reported that low levels of anti-IFN-γ autoAbs were found in plasma from healthy controls and patients with pulmonary mycobacterial infection but that these autoAbs were not biologically active in terms of inhibiting STAT-1 phosphorylation (7). Recent reports suggest that the neutralizing capacity is more important than the antibody concentration itself for determining the predisposition to a disease (13). Therefore, the identification of the neutralizing anti-IFN-γ autoAbs in individuals with disseminated tuberculous or late-onset NTM infection is crucial for identifying pathogenic autoAbs. Recently, the characteristics of anti-IFN-γ autoAbs were investigated and it was demonstrated that the anti-IFN-γ autoAbs in patients with unusual intracellular pathogens recognized the C-terminus of the IFN-γ linear epitope containing the KRKR motif and were mainly of the IgG1 and IgG4 subclasses (9). Even though those autoAbs had neutralizing activity, in some AOID patients, the autoAbs are unable to bind to this peptide. It is possible that those autoAbs bind to the other epitopes of IFN-γ.

The epitopes required for IFN-γ-mediated functions were either the receptor binding epitope or other regions that were identified by mAbs. In 1989, the mouse anti-IFN-γ mAb clone B27, which binds to the epitope formed by dimeric IFN-γ, showed strong neutralizing capacity (10). Recently, the conformational epitope recognized by neutralizing mAbs to human IFN-γ was identified using human-bovine IFN-γ chimeras (14). The mAbs that bind helical region E (IFN-γ_83−98_) had strong neutralizing capacity, whereas mAbs binding in helical region A (IFN-γ_1−18_), which is involved in IFN-γ receptor interaction, had less potent neutralizing capacity. These data suggested that the neutralizing capacity of anti-IFN-γ mAbs depends on their binding site in relation to conformational epitopes. Because the B27 mAb is a potent neutralizing antibody, the epitope recognized by this mAb should be a bioactive epitope. However, the exact epitope targeted by the B27 mAb was poorly defined. In this study, the neutralizing activity of the B27 mAb was confirmed by a cell-based assay measuring MHC class II expression on the cell surface. To identify which epitope the B27 mAb recognized, epitope mapping using 20 amino acid peptides from IFN-γ protein was performed. The results showed that B27 mAb did not bind the synthetic peptides but recognized the full-length IFN-γ. These data suggest that the B27 epitope is a conformation-dependent epitope that is crucial for IFN-γ bioactivity. This result corresponds with the previously reported finding that the B27 mAb recognized non-denatured IFN-γ (10).

To determine whether the B27 epitope was recognized by other neutralizing mAbs, the B133.5, and MD-1 mAbs were analyzed. In this study, the neutralizing capacity of these mAbs was confirmed by a cell-based functional assay and was compared with that of B27 mAb. The results demonstrated that these mAbs have less neutralizing capacity compared to that of the B27 mAb. In addition, the epitope that was recognized by these mAbs was clustered by epitope binning. Each pair of mAbs was analyzed, and the results showed that the B27 mAb inhibited the binding of the B133.5 mAb to IFN-γ, whereas the B133.5 mAb did not affect the B27 mAb binding. This phenomenon suggested that B27 binding contributes to allosteric changes in IFN-γ; therefore, the B133.5 mAb could not recognize IFN-γ. In addition, saturating IFN-γ with the B133.5 mAb blocks MD-1 binding and vice versa. It is assumed that the B133.5 and MD-1 mAbs bind the same epitope or overlapping epitopes. Similar to the B27 mAb, the B133.5, and MD-1 mAbs did not bind the synthetic peptides, suggesting that these mAbs recognized a discontinuous epitope of IFN-γ. Because the binding affinity of the B27, B133.5, and MD-1 mAbs by kinetic analysis was comparable, it is presumed that the neutralizing capacity of these mAbs is based on their binding region.

In AOID patients, the clinical significance of B27 epitope recognition has never been reported. Our study attempted to characterize the autoAbs in AOID patients to identify pathogenic autoAbs that contribute to severe opportunistic infections. A competitive-binding ELISA was developed to detect B27 epitope-binding autoAbs. Furthermore, B133.5, and MD-1 epitope recognizing autoAbs were characterized to identify the clone and pattern of the anti- IFN-γ autoAbs in patients. The clone of an autoAb that recognizes the same or adjacent epitopes to the B27, B133.5, and MD-1 mAbs is presumed to be a neutralizing antibody. In this study, nine AOID patients (A1–A9) were included. Patient sera obtained from three subjects (patients A2, A3, and A6) displayed ~60% inhibition with all mAbs used in this study. These patient sera had a high relative level of anti-IFN-γ autoAbs and a high degree of neutralizing capacity, which were detected by indirect ELISA and flow cytometry, respectively. In this group, the IFN-γ-mediated signaling in patients was inhibited by the various clones of the neutralizing autoAbs that recognized the B27, B133.5, and MD-1 epitopes.

The level of total anti-IFN-γ autoAbs did not always correlate with the neutralizing capacity. Patient sera from three subjects (A1, A4, and A9) had similar levels of anti-IFN-γ autoAbs but showed different degrees of neutralization. The autoAbs in the A1 and A4 sera did not show neutralizing activity in the cell-based assay. Among the neutralizing autoAbs detected by the competitive-binding ELISA, the serum obtained from A1 showed ~20% inhibition with the three mAbs. The serum obtained from A4 displayed ~40% competition with the B133.5 mAb but had <20% inhibition with the other mAbs. Interestingly, the serum from A9 had neutralizing capacity. Based on the competitive-binding ELISA, the A9 serum exhibited ~60% inhibition of B133.5 and MD-1, which was comparable with the serum obtained from patients A2, A3, and A6, but this serum showed ~30% inhibition of the B27 mAb. Although the A9 serum had neutralizing activity, the neutralizing capacity was less than those in the A2, A3, and A6 sera. These results suggest a crucial role for B27 in epitope-recognizing autoAbs in patients. In the latter group, the patient sera with low levels of anti-IFN-γ autoAbs (A5, A7, and A8) had no neutralizing ability and exhibited very low competition with B27 mAb. Although the patient serum from A5 and A7 could compete with B133.5 and MD-1 by ~30–40%, those autoAbs did not neutralize IFN-γ. Taken together, the B27 epitope-recognizing autoAbs were present in the AOID patient sera.

Previously, the IFN-γ C-terminal region, which contains the KRKR motif, has been reported as the epitope that is recognized by the anti-IFN-γ autoAbs in patients with opportunistic infections (7, 9). This region has been reported to be involved in IFN-γ receptor activation. However, the binding of this region to the receptor has not been confirmed by X-ray crystallography (15). Surprisingly, in some patients, their autoAbs did not bind to this region (8, 9). This observation was confirmed by our study, and the epitope mapping of the human anti-IFN-γ autoAbs in the serum of an AOID patient (A3) that used synthetic peptides showed that the autoAbs could recognize both peptide 14 and peptide 15 which share the KRKR motif. Remarkably, in other patient sera that had high neutralizing activity (A2 and A6), the autoAbs did not recognize this peptide. These data suggest that the neutralizing autoAbs in these patients react with discontinuous epitopes located outside of the KRKR motif-containing region.

Currently, there are many methods that have been described for detecting anti-IFN-γ autoAbs. Total anti-IFN-γ autoAbs were detected by indirect ELISA, western blot analysis, or a particle-based technique (4, 16, 17). However, these methods cannot evaluate the neutralizing activity. Most commonly, the neutralizing capacity of anti-IFN-γ autoAbs is measured by a functional, cell-based assay that measures pSTAT1 by flow cytometry (4, 7, 11). A recent publication reported using the QuantiFERON TB Gold in-tube test (QFT-GIT), a commercial IFN-γ release assay used for clinical tuberculosis (TB) testing, for the screening of neutralizing anti-IFN-γ autoAbs in patient sera (18, 19). With this method, the IFN-γ level secreted from TB antigen- or mitogen-stimulated cells was detected by ELISA. In the presence of anti-IFN-γ autoAbs in patient sera, IFN-γ was undetectable or had extremely low IFN-γ levels due to inhibition by these autoAbs, resulting in an indeterminate QFT-GIT. Based on an inhibition assay similar to QFT-GIT, it has been reported that there is no relationship between the level of neutralizing anti-IFN-γ autoAbs and disease course (20). This might have been caused by an indeterminate result that did not absolutely reflect the neutralizing autoAbs. According to our established assay, the autoAb population that recognizing the corresponding domain of neutralizing mouse anti- IFN-γ mAbs were demonstrated. The correlation of these autoAb population with the disease activity should be further investigated.

Because the clinical manifestations of patients with disseminated NTM are non-specific, the assessment of the neutralizing anti-IFN-γ autoAbs will be useful for diagnosis, prognosis, and therapeutic interventions. In these patients, treatment with IFN-γ fails to ameliorate the symptoms because neutralizing anti-IFN-γ Abs block IFN-γ-mediated functions (21). On the other hand, an anti-CD20 antibody, rituximab, has been used for the treatment of patients with disseminated NTM infection who had anti-IFN-γ autoAbs (22–24). The rituximab treatment was found to restore IFN-γ signaling and improve symptoms. In addition, long-term antimicrobial therapy is required for combined treatment. Prognosis in disseminated NTM patients with neutralizing anti-IFN-γ autoAbs is more favorable than prognosis in disseminated NTM patients with HIV infection or without neutralizing anti-IFN-γ autoAbs (13). Therefore, evaluating the neutralizing autoAbs is helpful for identifying the proper therapeutic interventions to improve the survival rate of these patients. In addition to anti-IFN-γ autoAbs, the neutralizing capacity of autoAbs with regard to other cytokines, such as granulocyte macrophage-colony stimulating factor (GM-CSF) or type I IFN, which mediate the susceptibility to intracellular pathogens in previously healthy adults should be taken into consideration. The approach used in this study could be applied to develop a method for evaluating the binding location of those autoAbs. Regarding future directions, the correlation between the neutralizing autoAbs, and disease progression/therapeutic interventions should be investigated.

In conclusion, this study demonstrated that the anti-IFN-γ autoAbs in AOID patients recognized a discontinuous epitope of IFN-γ. With the development of a competitive-binding ELISA, the binding domains of certain autoAbs were identified. An understanding of the role of the crucial antigenic determinants that are recognized by pathogenic autoAbs will provide a foundation for clinical intervention and efficient therapeutic strategies.

## Data Availability

All datasets generated for this study are included in the manuscript and/or the Supplementary Files.

## Ethics Statement

All subjects gave written informed consent in accordance with the Declaration of Helsinki. The protocol was approved by the Human Experimentation Committee, Research Institute for Health Sciences, Chiang Mai University, Chiang Mai, Thailand.

## Author Contributions

UY and WT designed and performed the experiments, analyzed the data, and wrote the manuscript. SM generated the recombinant IFN-γ. SS and JW provided the patient samples and suggestions. RC, JP, and KS collected and provided the patient samples. EL performed the BLI experiments. KC performed the pSTAT1 analysis. CT supervised this study, provided the critical suggestions and discussions, and revised the manuscript.

### Conflict of Interest Statement

EL was employed by company Pall Filtration. The remaining authors declare that the research was conducted in the absence of any commercial or financial relationships that could be construed as a potential conflict of interest.
