# Supplementary material for: Neutralizing Activity of Anti-interferon-γ Autoantibodies in Adult-Onset Immunodeficiency Is Associated With Their Binding Domains
Source: Front Immunol. 2019 Aug 14;10:1905. doi: 10.3389/fimmu.2019.01905 (PMC6702949; doi:10.3389/fimmu.2019.01905)
Supplement: Supplementary file 1 [file Image_1.pdf]

Supplementary Material

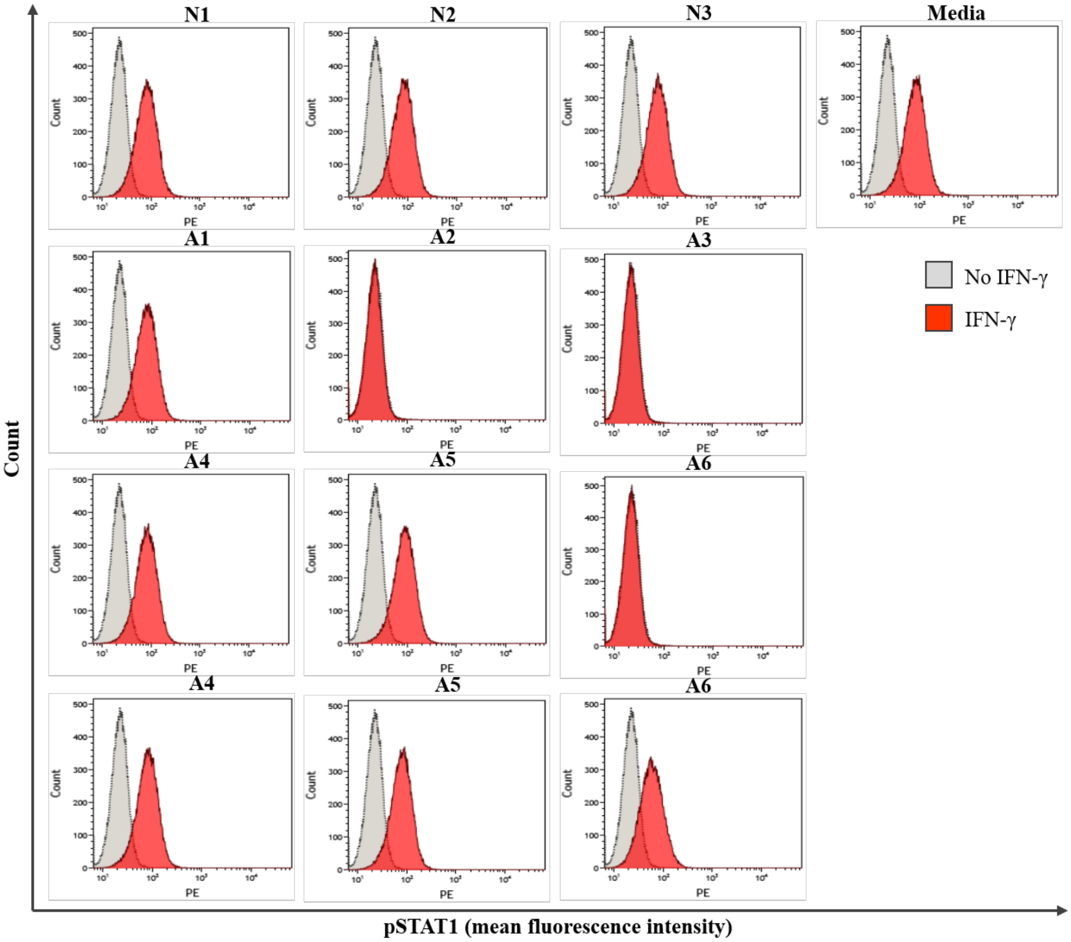

Supplementary Figure 1. STAT1 phosphorylation in THP-1 cells after IFN-γ treatment. THP-1 cells were treated with rIFN-γ in the presence or absence of patient serum or healthy serum. The intracellular pSTAT1 level was evaluated by flow cytometry. Media control was used as system control. N, normal serum (n=3); A, AOID patient serum (n=9).
